# A near-chromosome level genome assembly of the European hoverfly, *Sphaerophoria rueppellii* (Diptera: Syrphidae), provides comparative insights into insecticide resistance-related gene family evolution

**DOI:** 10.1186/s12864-022-08436-5

**Published:** 2022-03-12

**Authors:** Emma Bailey, Linda Field, Christopher Rawlings, Rob King, Fady Mohareb, Keywan-Hassani Pak, David Hughes, Martin Williamson, Eric Ganko, Benjamin Buer, Ralf Nauen

**Affiliations:** 1 Department of Biointeractions and Crop Protection, Rothamsted Research, Harpenden, UK; 2 Department of Computational and Analytical Sciences, Rothamsted Research, Harpenden, UK; 3 The Bioinformatics Group, Cranfield Soil and Agrifood Institute, Cranfield University, Cranfield, UK; 4 Seeds Research, Syngenta Crop Protection, LLC, Research Triangle Park, Durham, NC USA; 5 Bayer AG, Crop Science Division, R&D, Monheim, Germany

**Keywords:** *Sphaerophoria rueppellii*, Hoverfly, PacBio, Illumina, Hi-C, Whole genome sequencing, Beneficial predator, Insecticide resistance, Comparative genomics, Diptera, Syrphidae, Crop pests

## Abstract

**Background:**

*Sphaerophoria rueppellii*, a European species of hoverfly, is a highly effective beneficial predator of hemipteran crop pests including aphids, thrips and coleopteran/lepidopteran larvae in integrated pest management (IPM) programmes. It is also a key pollinator of a wide variety of important agricultural crops. No genomic information is currently available for *S. rueppellii*. Without genomic information for such beneficial predator species, we are unable to perform comparative analyses of insecticide target-sites and genes encoding metabolic enzymes potentially responsible for insecticide resistance, between crop pests and their predators. These metabolic mechanisms include several gene families - cytochrome P450 monooxygenases (P450s), ATP binding cassette transporters (ABCs), glutathione-*S*-transferases (GSTs), UDP-glycosyltransferases (UGTs) and carboxyl/choline esterases (CCEs).

**Methods and findings:**

In this study, a high-quality near-chromosome level *de novo* genome assembly (as well as a mitochondrial genome assembly) for *S. rueppellii* has been generated using a hybrid approach with PacBio long-read and Illumina short-read data, followed by super scaffolding using Hi-C data. The final assembly achieved a scaffold N50 of 87Mb, a total genome size of 537.6Mb and a level of completeness of 96% using a set of 1,658 core insect genes present as full-length genes. The assembly was annotated with 14,249 protein-coding genes. Comparative analysis revealed gene expansions of CYP6Zx P450s, epsilon-class GSTs, dietary CCEs and multiple UGT families (UGT37/302/308/430/431). Conversely, ABCs, delta-class GSTs and non-CYP6Zx P450s showed limited expansion. Differences were seen in the distributions of resistance-associated gene families across subfamilies between *S. rueppellii* and some hemipteran crop pests. Additionally, *S. rueppellii* had larger numbers of detoxification genes than other pollinator species.

**Conclusion and significance:**

This assembly is the first published genome for a predatory member of the Syrphidae family and will serve as a useful resource for further research into selectivity and potential tolerance of insecticides by beneficial predators. Furthermore, the expansion of some gene families often linked to insecticide resistance and selectivity may be an indicator of the capacity of this predator to detoxify IPM selective insecticides. These findings could be exploited by targeted insecticide screens and functional studies to increase effectiveness of IPM strategies, which aim to increase crop yields by sustainably and effectively controlling pests without impacting beneficial predator populations.

**Supplementary Information:**

The online version contains supplementary material available at 10.1186/s12864-022-08436-5.

## Introduction

Loss of crops to insect pests can account for more than 10% of potential yield, as a result of both direct feeding damage and the transfer of plant viruses via insect feeding [1]. Methods of controlling insect pests are therefore critical to ensure that crop yields are maximised to sustain the growing world population. Insecticides play a key role in pest control strategies. Many modern insecticides are known to be selective for pests without harming beneficial predators. However, some insecticides such as pyrethroids tend to be non-specific and as a result are often toxic to both their target pest species and beneficial predators. Applications of such non-specific insecticides can reduce predator populations so that they are unable to act as an effective natural control. This can lead to pest populations surging, with instances of higher populations than pre-pesticide application [2–4].

Hoverflies (Diptera: Syrphidae), such as *Sphaerophoria rueppellii* which is native to Europe and Mediterranean counties, are effective in the biological control of crop pests. Syrphid adults typically feed on nectar and pollen, however, the larvae of roughly one-third of syrphid species feed on crop pests such as aphids, thrips and coleopteran and lepidopteran larvae [5–11]. Predatory Syrphidae are able to feed on up to ~500 aphids during their larval stage, which is a higher daily feeding rate than other aphid predators [12]. For example, *S. rueppellii* were able to reduce aphid (*Myzus persicae)* populations by 84% in a field experiment [13]. Specialised adaptations present within adult female Syrphidae allow them to detect aphid pheromones and increase their efficacy as biological control agents. Adult females often lay their eggs in close proximity to aphid colonies to ensure a plentiful food supply for emerging larvae [14]. Syrphid adults also avoid laying their eggs close to parasitised aphids [15] which reduces intraguild predation between parasitoids and hoverflies and thus allows for them to be safely combined in IPM strategies. Such strategies can result in more effective pest control compared to using only one beneficial predator species, especially when attempting to control multiple species of pest [16]. Overall, it is unsurprising that Syrphidae are considered to be amongst the most important aphid predators and a key tool for biological control [17, 18].

Alongside pest control, adult hoverflies play a key role in pollination [19] and are considered the second most important pollinator after the Apidae bee families [20]. Unlike bees, hoverflies are highly migratory and therefore capable of transporting pollen over long distances, which has benefits for both the plants and other non-migratory pollinators [21]. Pollination experiments have shown that hoverflies increase seed number in food crops such as strawberry, oilseed rape and sweet pepper (which also showed increased fruit abundance) [13, 22, 23].

This dual role as effective pollinators and biological control agents [11] makes hoverflies hugely attractive for commercial use and also highlights the need to develop IPM strategies which conserve their populations. The aim of this work was to produce a high-quality genome assembly for *S. rueppellii*, to serve as a resource for research into this species as well as the wider Syrphidae family. This family consists of ~6000 species worldwide [19, 24] and is therefore a potentially valuable source of biological control agents.

The number of sequenced beneficial predator genomes has been trailing behind crop pest genomes in recent years, although numbers are now on the rise, especially with the progress being made by the Darwin tree of Life (DToL) sequencing project [25]. High quality genomes have already been released by DToL for some beneficial predators such as green lacewing (*Chrysoperla carnea*) and the seven spotted ladybird (*Coccinella septempunctata*). Other publicly available beneficial predator genomes include: a phytoseiid mite, parasitoid wasps, a minute pirate bug and lady beetles [26–29]. To date the only available genome for the Syrphidae family is the non-predatory European hoverfly (*Eristalis pertinax*) released by DToL (but not yet annotated at the time of writing). So the *S. rueppellii* genome is the first available for a predatory member of the Syrphidae family.

The EU Directive on Sustainable Use of Pesticides 2009/128/EC [30] means that IPM strategies, including the use of beneficial predators [31–35], are growing in necessity. These strategies can be supported by comparative analyses of the genomes of predators and pests, focusing on potential differences in insecticide tolerance mechanisms based on both target-site selectivity and metabolism.

There are two main types of insecticide resistance mechanisms: mutations in insecticide target genes that prevent the insecticide binding to the target [36] and duplication or increased expression of genes encoding enzymes which can metabolise insecticides. Gene families associated with metabolic resistance include cytochrome P450 monooxygenases (P450s), ATP binding cassette transporters (ABCs), glutathione S-transferases (GSTs), UDP-glucosyltransferases (UGTs) and carboxyl/choline esterases (CCEs) [37–42]. Comparisons of these mechanisms in beneficial predators and crop pests could help identify insecticides which will target crop pests but have limited impact on beneficial predator populations. This information could prove key to developing successful IPM strategies which exploit differences in insecticide selectivity between the predator and crop pests. Improving the availability of beneficial predator genomes could also aid the selection of beneficial predators with genes/mutations for insecticide resistance before being released in the field for biological control [43].

The results presented here provide a comprehensive foundation for further study of insecticide tolerance and selectivity mechanisms in beneficial predators and how they compare to crop pests.

## Materials and methods

### Sample preparation and sequencing

*S. rueppellii* larvae were obtained from ‘biopestgroup.com’. CO_2_ was used for anaesthesia to allow the insects to be sorted from the substrate. The larvae were then flash frozen with liquid N_2_ and stored at -80°C. The whole process was completed within 48 hours of arrival.

For transcriptome sequencing, RNA extractions were carried out in-house at Rothamsted Research using the Bioline Isolate II RNA Mini Kit. 30μg of RNA was obtained from 5 individuals. The library was constructed with an insert size of 150bp and PolyA selection for rRNA removal. Sequencing was performed by Genewiz (New Jersey, US) using Illumina HiSeq 4000 with a 2x150bp paired-end configuration.

For short-read genomic sequencing, DNA extractions were performed in-house at Rothamsted Research using the commercial DNAzol reagent. Short reads were sequenced using 1.1μg of DNA obtained from 5 individuals and a library with an insert size of 200bp. Sequencing was performed by Genewiz (New Jersey, US) using Illumina HiSeq 4000 with a 2x150bp paired-end configuration. K-mer counting of the raw Illumina DNA data was performed using Jellyfish 2.2.6 [44]. Canonical (-C) 21-mers (-m 21) were counted and a histogram of k-mer frequencies outputted. GenomeScope 2.0 [45] was used to process this histogram with ploidy set to 2 and maximum k-mer coverage cut-off set to 10,000.

For long-read genomic sequencing, whole insects were sent directly to Georgia Genomics (University of Georgia, US) who performed the DNA extractions using ~15 individuals. To obtain long-read PacBio data, a 15-30Kb SMRTbell library was produced with an insert size of 24,000bp and a 15 hour sequencing run was carried out using PacBio Sequel II.

For Hi-C sequencing, whole insects were sent directly to Arima Genomics (San Diego, US) who carried out the DNA extractions using 10 individuals. Arima-QC and library preparation were also performed in-house. Sequencing was performed using Illumina HiSeq X with a 2 x 150bp paired-end configuration.

### Genome quality assessment

To evaluate the redundancy of the final assembly, short-read Illumina data was mapped back to the final genome using BWA-MEM [46]. Samtools-flagstat [47] was used to assess mapping rates. To assess read depth distribution, bamCoverage from deepTools [48] was used to produce a bigWig coverage track.

Basic metrics from the genome assembly were calculated using a script developed for the ‘Assemblathon’ [49]. These metrics include scaffold/contig N50, longest and shortest scaffold length, number of scaffolds exceeding a range of lengths and number of gaps/N’s in the assembly.

The completeness of the genome assembly and annotation for *S. rueppellii* was assessed using the Benchmarking Universal Single-Copy Orthologs (BUSCO) [50] of the insect gene set (insecta odb9). ‘Genome’ mode was used to assess the assembly, and ‘protein’ mode to assess the annotation. ‘Fly’ was used as the training species for Augustus gene prediction. BUSCO assessments were then run with default parameters.

### *De novo* genome assembly

FastQC v.0.11.8 [51] was used to perform quality checks on the raw Illumina HiSeq DNA and RNA sequence data. Adapters were trimmed, low-quality bases (below a score of 3) were removed from the start and end of reads and any reads with a length less than 36 bases were also removed. Trimmomatic v.0.38 [52] was used for these trimming steps.

GenomeScope 2.0 [45] was used to perform k-mer analysis of Illumina short-reads with default parameters. The results were used to estimate genome size and get an indication of heterozygosity.

The raw PacBio reads were subsetted using a ‘SelectLongestReads’ script from: https://github.com/yechengxi/AssemblyUtility to reduce coverage from 277x to 150x coverage prior to assembly. The subsetted PacBio long reads were then assembled into contigs with the Flye v2.5. *de novo* assembler [53, 54] with the following parameters: ‘--genome-size 300m -i 3 --meta’. This subsetting was used to reduce duplication in the assembly outputted by Flye whilst maintaining the completeness of the genome.

The subsetted PacBio long-reads and Illumina DNA short reads were also assembled into contigs using Platanus Allee v2.2.2 [55] with default parameters. This is a hybrid assembler designed for heterozygous data.

QuickMerge v0.3 [56] was used to merge the Flye and Platanus-Allee assemblies, with Flye as the reference assembly. BUSCO outputs were compared between the merged assembly and the standalone assemblies to identify core insect genes which had been lost during the merging process. Full-length contigs containing these missing genes were extracted from the standalone assemblies and added to the merged assembly, based on the assumption that these contigs would also contain other missing genes (i.e. those not included in BUSCO’s list of 1,658 core insect genes).

Purge Haplotigs v1.0.0 [57] was next used to perform redundant contig removal from the merged assembly. Parameters ‘-l 5 -m 30 -h 190’ were chosen from the coverage histogram outputted in the first step of the pipeline. The percent cutoff for identifying a contig as a haplotig was set to ‘-a 40’, (the default value is 70, however a lower cutoff was chosen due to a very high level of duplication). This tool takes read depth coverage into consideration to reduce over-purging of repetitive regions and paralogous contigs, whilst still coping well with highly heterozygous assemblies.

The Hi-C data was processed using Juicer v1.5 [58] and used as input to the 3D-DNA de novo genome assembly pipeline (version 180922) [59] alongside the draft assembly to produce a candidate chromosome-length genome assembly. Contact matrices were generated by aligning the Hi-C dataset to the genome assembly after Hi-C scaffolding, and were then visualised using JuiceBox Assembly Tools v1.11.08 [60]. The parameters used were as follows: ‘--mode haploid --build-gapped-map --sort-output’. Additional finishing on the scaffolds was performed in JuiceBox to correct mis-joins.

Multiple rounds of Pilon [61] error polishing were performed, using the Illumina short read data, until no further improvement in BUSCO score was seen. A final round of Purge Haplotigs was then performed to reduce duplication further. Parameters ‘-l 10 -m 50 -h 150’ were chosen from the coverage histogram outputted in the first step of the pipeline. The percent cutoff for identifying a contig as a haplotig was set to ‘-a 80’.

### Mitochondrial genome assembly

The mitochondrial genome was found and extracted by running a BLAST search of the *S. rueppellii* genome against the *Syrphus ribesii* mitochondrial genome, which is publicly available at NCBI, GenBank accession number: MW091497.1.

### Annotation

Gene prediction was performed using the MAKER v2.31.8 pipeline [62] through the incorporation of both transcriptome evidence and *ab initio* gene prediction as well as a custom repeat library (see below). MAKER was run using Augustus v3.3.1 [63], GeneMark-ES v4.32 [64] and FGeneSH v8.0.0 [65] as well as EVidenceModeler v1.1.1 [66] with default masking options.

A *de novo* species specific repeat library was constructed using RepeatModeller v1.0.7 [67] to identify repeat models. These models were searched against the GenBank non-redundant (*nr*) protein database for Arthropoda (e value <10^-3^) using Blastx to remove any potential protein-coding genes. This was combined with transposon data to create a custom library. Transposons were identified from the transcriptome assembly by running HMMER: hmmscan [68] against the Pfam database [69] and filtering the resultant Pfam descriptions for those containing “transposon”. A search for transposons was also performed on transcripts produced from MAKER and these transposons were then added to the custom repeat library which was used for a second round of MAKER. RepeatMasker v4.0.7 [70] was used to mask repeats in the genome assembly using these repeat libraries, as well as to estimate the abundances of all predicted repeats.

RNA-seq reads were mapped to the genome with HISAT2 v2.0.5 [71] for assembly with StringTie v1.0.1 [72]. A de novo assembly was also done using Trinity v2.5.1 [73]. The best transcripts were selected from the Trinity and StringTie assemblies using Evigene v19.jan01 [74].

Evidence from assembled transcripts was transferred to the genome assembly via MAKER. The output from this was then used to produce a high confidence level gene model training set. Overlapping and redundant gene models were removed. Augustus and GeneMark were trained using this training set prior to being used for *ab initio* gene predictions. FGeneSH was run based on the *Drosophila melanogaster* genome.

The best transcripts (classified by reasonable transcript size and homology to other species) from both the *ab initio* gene prediction annotation and the transcriptome-based annotation were selected using Evigene and combined to create the final annotation.

*S. rueppellii* protein sequences were aligned using Blastp against the non-redundant (nr) NCBI protein database for Arthropoda. InterProscan searches were run against several databases (CDD, HAMAP, HMMPAnther, HMMPfam, HMMPIR, FPrintScan, BlastProDom, ProfileScan, HMMTigr) for functional annotation. BLAST2GO [75] was used to assign gene ontology (GO annotations). Infernal v1.1.2 [76] was used to predict and annotate non-coding RNAs.

The mitochondrial genome was annotated using MITOS2 [77] with reference database ‘RefSeq 89 Metazoa’ and genetic code ‘5 Invertebrate’.

### Comparative genomics and phylogenetic analysis

To produce the species tree, orthogroup gene trees were produced using OrthoFinder [78] and the tree was inferred from these using the STAG method [79].

In order to identify candidate insecticide resistance genes, the PFAM domains assigned to gene models during annotation (as described in the ‘Genome Annotation’ methods section) were used as follows: CCEs (PF00135/IPR002018), GSTs (IPR004045/PF02798), (IPR004046/PF00043), P450s (IPR001128/PF00067), ABCs (IPR003439/PF00005) and UGTs (IPR002213/PF00201). Proteins from UniProtKB for the classes of interest, from hemipteran species, were used for BLAST queries against *S. rueppellii* to identify any missed genes and to assist with subfamily assignment within these classes. Subfamily assignment for *S. rueppellii* gene families was finalised using phylogenetic trees which were produced using MAFFT alignments [80, 81] and RaxML v8.2.11 [82]. The GAMMA LG protein model [83] was used (MEGAX was used to determine the best substitution model [84]) and a bootstrap consensus tree was inferred from 100 replicates.

Manual checks and curation were performed for genes potentially involved in insecticide resistance. Increased copy numbers of genes linked to insecticide resistance often led to adjacent tandem duplications being incorrectly annotated by MAKER as one gene model; therefore curation was important to prevent incorrect gene numbers being reported in later analyses. The exon/intron boundaries and start/stop codons of the genes were confirmed through visualization in IGV [85] of RNAseq data mapped to the genome using HISAT2 v2.0.5 [71] and the gene models were edited in Geneious where necessary.

The P450s were classified and named by Dr David Nelson [86]. The UGTs were classified and named by Dr Michael Court [87]. Nomenclature of P450s and UGTs is based on the evolutionary relationships of the sequences. P450 and UGT sequences were BLAST searched against named insect sequences and were assigned to known families if they were >40% (for P450 families) or >45% (for UGT families) identical. Other sequences were assigned to new families based on their clustering on trees and their percent identity to each other.

## Results and discussion

### Sequencing

~30 individuals of *S. rueppellii* were required to produce sufficient DNA and RNA for sequencing. Since they were obtained commercially, the level of inbreeding of the culture was not known. However, all individuals were obtained from a single colony within the rearing facility. A high heterozygosity level was therefore a possibility and this was kept in mind during the assembly process.

The DNA sequencing generated 6,748,327 PacBio reads with a total length of 83.2 Gbp (277x) and a polymerase read length N50 of 63,285bp.

A total of 125.3Gb of sequencing data (417,662,063 reads) was produced from the Illumina HiSeq platform for whole genome sequencing, as well as 36.9Gb (123,298,454 reads) for transcriptome sequencing. Quality trimming of Illumina reads using Trimmomatic to remove adapters and any reads <36bp resulted in 405,634,072 reads for whole genome sequencing and 116,917,664 reads for transcriptome sequencing.

A total of 21.6Gb of sequencing data was produced from Arima-HiC. Analysis of proximal ligation gave a library QC metric of 30% (a high-quality Arima-HiC library is >15%).

### Genome metrics evaluation based on raw reads

**Fig. 1:**
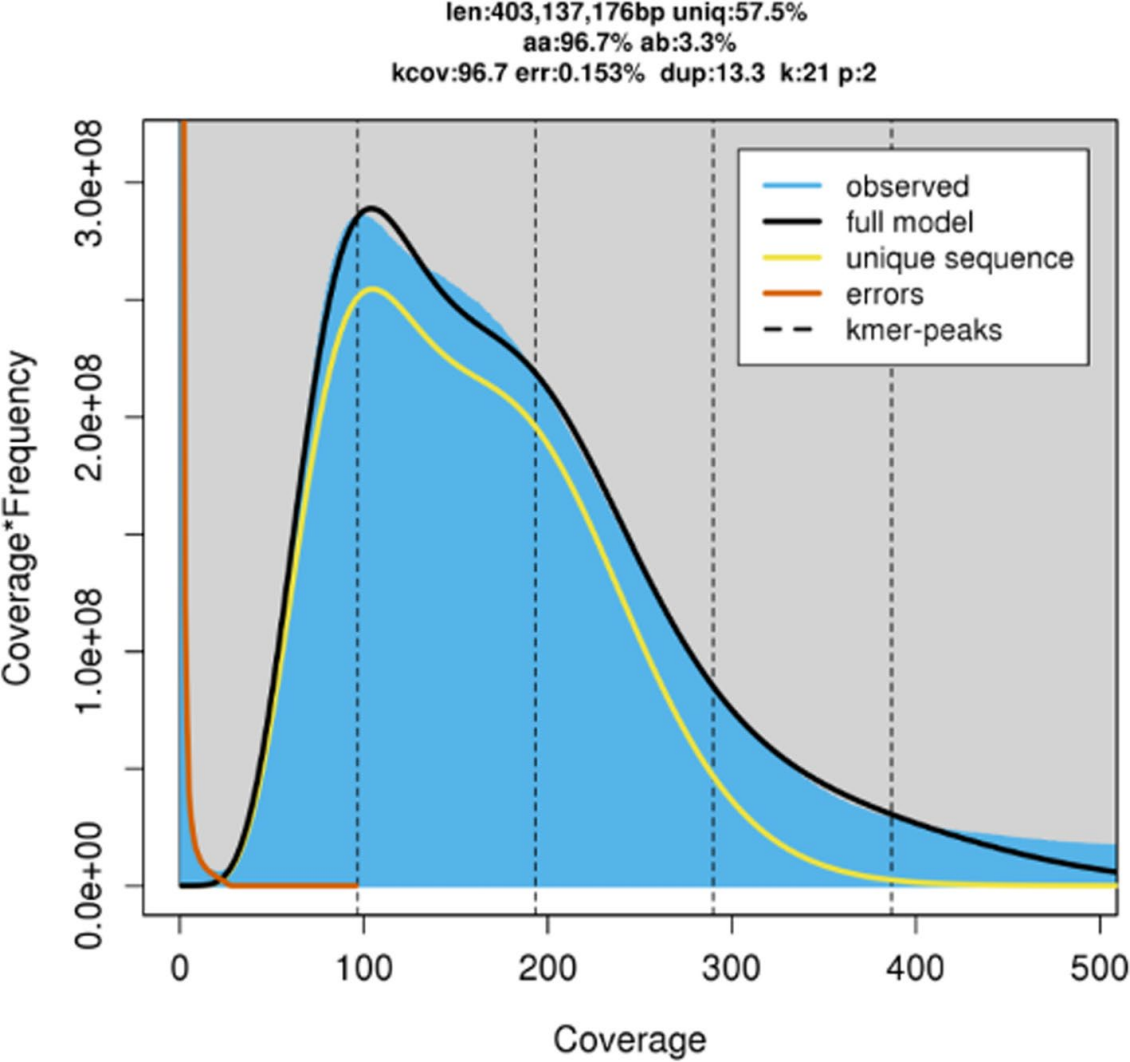
GenomeScope v2.0 k-mer profile plot for the *S. rueppellii* genome, based on 21-mers in Illumina reads. The observed k-mer frequency distribution is depicted in blue, whereas the GenomeScope fitmodel is shown as a black line. The unique and putative error k-mer distributions are plotted in yellow and red, respectively

The raw read k-mer analysis with GenomeScope 2.0 (see Fig. 1) estimated a haploid genome size of 403Mb, which is an underestimate of the final assembly size of 537Mb. However, such discrepancies are often seen when using k-mer frequency to estimate genome size in genomes with high repeat content and high heterozygosity [88]. Genome repeat length was 170Mb, 42% of the total estimated genome size. The heterozygosity rate ranged from 3.24% to 3.36%. This indicates a fairly high level of heterozygosity, which was taken into consideration in the assembly strategy.

### Assembly

Several assemblers were trialed to generate the assembly (including Canu [89], DBG2OLC [90] and wtdbg2 [91]), however, many struggled to produce a good quality assembly, perhaps due to the high repeat content and heterozygosity of the genome. Flye and Platanus-Allee produced the best quality assemblies. Flye had the best assembly statistics in terms of scaffold N50 (100,207bp with 18 scaffolds >1 million bp) and BUSCO completeness score (99.2%). However, duplication was very high (48.3%) for this assembly, even after subsetting the longest reads to get 150x coverage (duplication was 63.8% prior to subsetting). The total number of scaffolds was 50,164. Platanus-Allee had a lower scaffold N50 (42,845bp with 0 scaffolds >1 million bp) and a slightly lower BUSCO completeness score (97.6%), but duplication was much lower (3.6%). The total number of scaffolds was 67,142.

In order to retain the high contiguity of the Flye assembly, whilst attempting to reduce its high duplication percentage, the Flye and Platanus-Allee assemblies were merged using QuickMerge. Some manual curation was also performed to bring back falsely removed contigs. This resulted in an assembly with a slightly lower completeness score of 96.5%, however, the duplication was reduced to 15.5% whilst preserving most of the long-length scaffolds produced using Flye. The assembly had a scaffold N50 of 67,653bp and a total of 59,284 scaffolds, 16 of which were >1 million bp.

A subsequent round of Purge Haplotigs brought the duplication score down to 4.6% whilst still maintaining a completeness of 95.6%. Scaffold N50 increased to 126,450bp and the total number of scaffolds was reduced to 15,009.

**Fig. 2:**
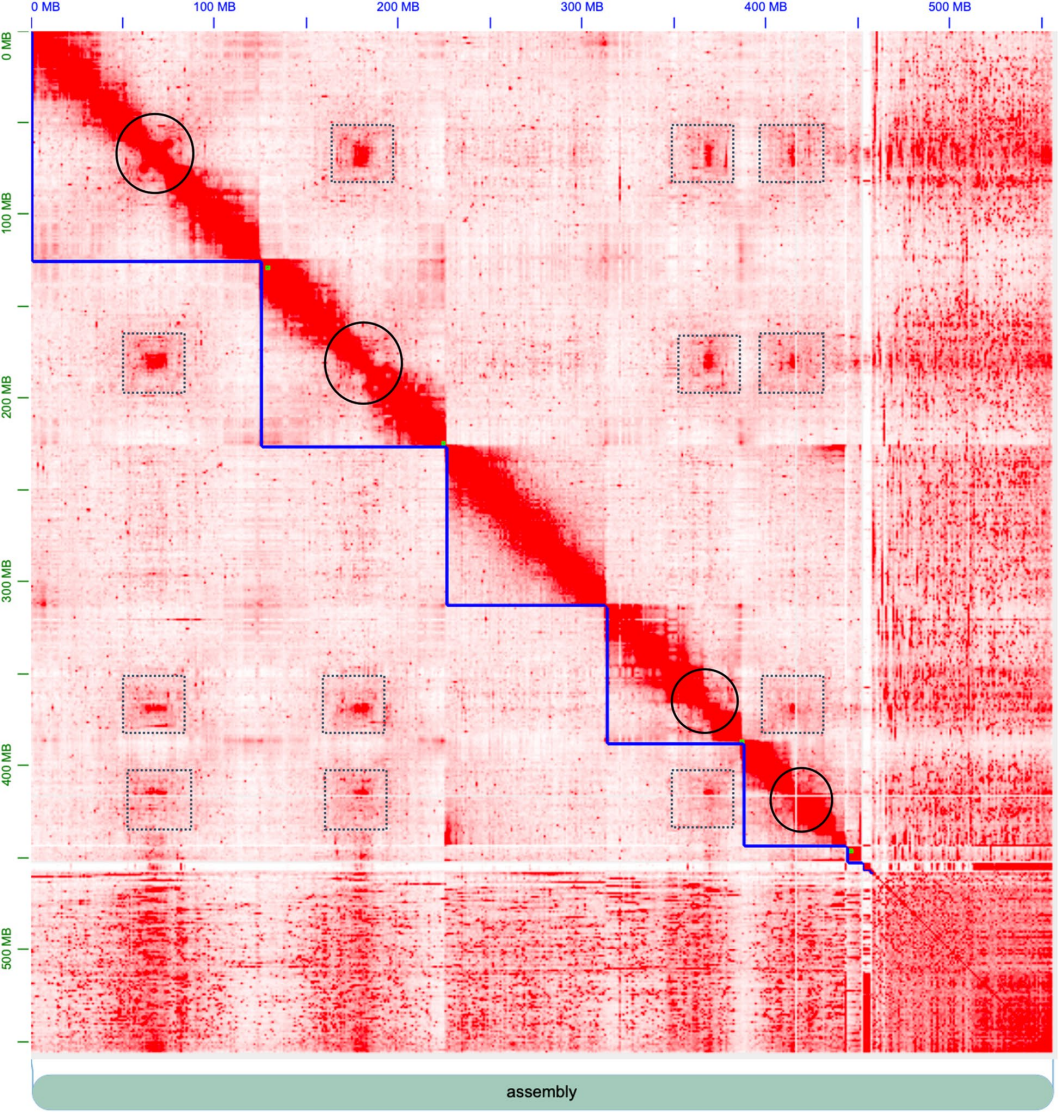
The *Sphaerophoria rueppellii* genome visualised in JuiceBox, with Hi-C contacts shown in red. Blue edges = superscaffolds/chromosomes. Black circles = likely centromeres. Grey boxes = centromere - centromere inter-chromosomal interactions. (Potential chromosome 3 had no obvious centromere, which may have been due to a mis-assembly. The 7th scaffold was mostly repeat regions - evidenced by the lack of interactions with the rest of the genome)

This draft assembly was next used for scaffolding with Hi-C data using the 3D-DNA *de novo* genome assembly pipeline. This increased the scaffold N50 to 87,361,475 bp, with 5 scaffolds > 10 million bp. The total number of scaffolds was reduced to 11,549, with 6 chromosomal-level scaffolds, numbered by sequence length (Fig. 2). There is currently no karyotypic information for *S. rueppellii* to confirm the correct number of chromosomes, however, this value corresponds to a cytogenetic analysis of *Eristalis tenax* which had 6 chromosomes [92]. The BUSCO completeness score was reduced to 94.6%, however, a round of Pilon error polishing brought this back up to 96.4% (subsequent rounds of Pilon worsened the BUSCO score). A final run with Purge Haplotigs reduced duplication from 4% to 3%. Statistics of the final assembly are shown in Table 1. The final assembly is available under accession GCA_920937365.1.

**Table 1:** Final assembly statistics for the *S. rueppellii* genome

**Number of scaffolds**	8,476
**Total size of scaffolds**	537,631,316 bp
**Longest scaffold**	125,413,692 bp
**Shortest scaffold**	957 bp
**Number of scaffolds > 1K bp**	8,412 (99.2%)
**Number of scaffolds > 10K bp**	2,095 (24.7%)
**Number of scaffolds > 100K bp**	70 (0.8%)
**Number of scaffolds > 1M bp**	9 (0.1%)
**Number of scaffolds > 10M bp**	5 (0.1%)
**N50 scaffold length**	87,097,991 bp
**Number of N’s**	56,988,920
**BUSCO**	C:96.0%[S:93.0%,D:3.0%], F:1.2%,M:2.8%

The final assembly size of 537.6Mb was slightly larger than the assembled genome size for *E. pertinax* (482Mb) [93], but closely matches the genome size for *Episyrphus balteatus* (530Mb) from the Syrphidae family, which was calculated using flow cytometry and can therefore be considered a more accurate estimate [94].

To further assess genome quality, the Illumina sequencing data was aligned back to the final genome to assess mapping rates and read depth distribution. Statistics are included in Table S1. 98.8% of reads were mapped, suggesting the genome is largely complete, with little novel sequence missing. 75% of reads were uniquely mapped, suggesting 25% of the genome is either repeat content or redundant, however, based on other Diptera genomes, 25% is a realistic value for repeat content [95]. The read depth distribution was fairly consistent across the genome, with the few high coverage/repetitive regions generally extraneous to the 6 chromosomal-level scaffolds (Fig. S1).

### Annotation

Gene prediction with MAKER identified 14,249 protein-coding genes with the proteins having a mean length of 465 amino acids. Of these, 10,789 (76%) had a match to NCBI’s non-redundant (*nr*) database and 12,000 (84%) contained InterPro motifs, domains or signatures. The longest protein found was a ‘nesprin-1 isoform’ at 17,083aa. The final proteome had a BUSCO completeness score of 87.3% (with 4.9% duplication).

From the Infernal tool inference of RNA alignments, a total of 2,292 non-coding RNA elements were found in the genome (Table S2). Transposable and repetitive elements made up 30% of the *S. rueppellii* genome (Table S3). This is consistent with previously reported repeat contents of Diptera genomes, which range widely from 7% (*Drosophila simulans*) to 55% (*Aedes aegypti*) [95]. 16.15% of the *S. rueppellii* genome (77,619,601bp) was masked for annotation - some repeats were annotated but not masked, such as those less than 10bp in length. The majority of these were LINES (9.97%) and interspersed repeats (14.35%).

### Mitochondria

**Fig. 3:**
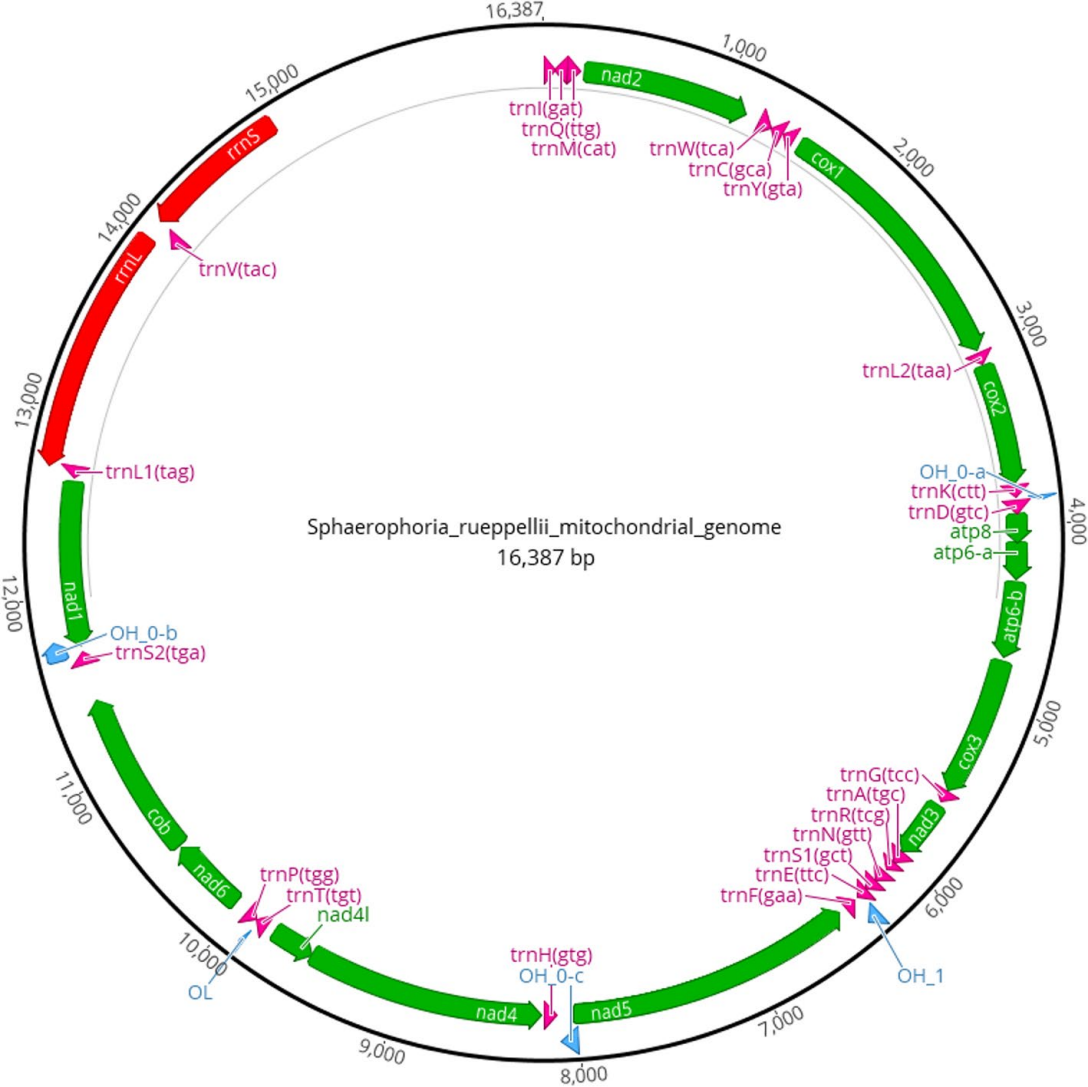
The mitochondrial genome for *Sphaerophoria rueppellii*, visualised using Geneious and annotation track obtained using MITOS2

The circularized mitochondrial genome of *S. rueppellii* was 16,387bp long. Annotation using MITOS2, identified 13 protein coding genes, 22 tRNA genes, 2 rRNA genes and an A+T rich region with a length of 1,500bp (Fig. 3). This composition is very similar to the *Syrphus ribesii* mitochondrial genome which is 16,530bp in length and also has 13 protein coding genes, 22 tRNA genes, 2 rRNA genes and an A-T rich region [96].

### Phylogeny

**Fig. 4:**
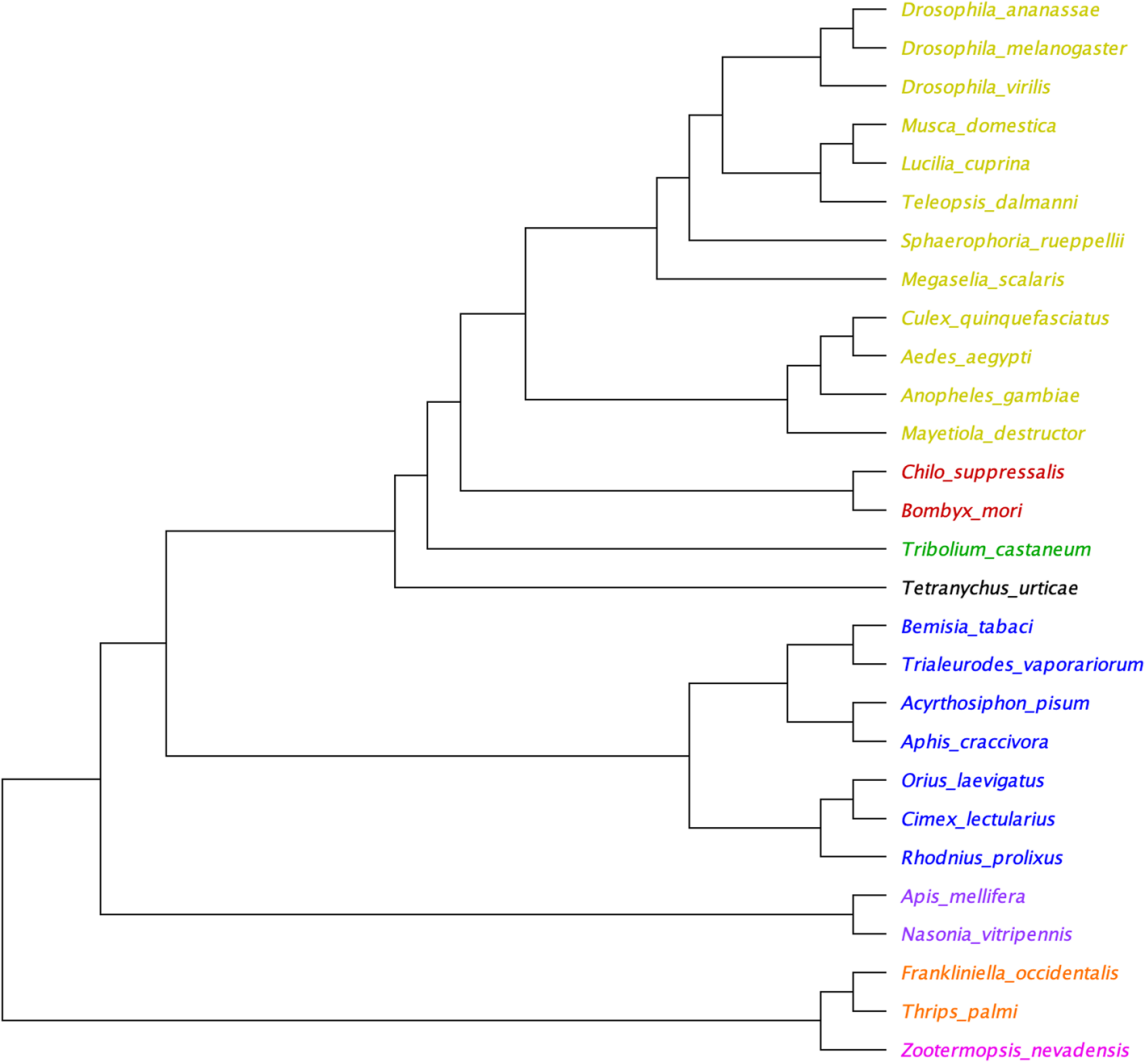
Phylogeny of Insecta, including crop pests and other beneficial predators

OrthoFinder assigned 435,592 genes (93.6% of total) to 28,834 orthogroups. There were 1,805 orthogroups with all species present and one of these consisted entirely of single-copy genes. Phylogenetic analysis correctly clustered *S. rueppellii* within the dipteran clade, between the Phoridae and Drosophilae families [97] (Fig. 4).

Species tree inferred using the STAG method. Nodes are coloured by order, yellow=Diptera, red=Lepidoptera, green=Coleoptera, black=Chelicerata, blue=Hemiptera, purple=Hymenoptera, orange=Thysanoptera, pink=Isoptera. Produced using the STAG tree inference method and full proteomes of the following species: *D. ananassae:* PRJNA12651, *D. melanogaster:* PRJNA13812, *D. virilis:* PRJNA12688, *M. domestica:* PRJNA176013, *L. cuprina:* PRJNA248412, *T. dalmanni:* PRJNA391339, *S. rueppellii:* (this study), *M. scalaris:* PRJEB1273, *C. quinquefasciatus:* PRJNA18751, *A. aegypti:* PRJNA318737, *A. gambiae:* PRJNA1438, *M. destructor:* PRJNA45867, *C. suppressalis*: PRJNA506136, *B. mori:* PRJNA205630, *T. castaneum*: PRJNA12540, *T. urticae:* PRJNA315122, *B. tabaci*: PRJNA312470, *T. vaporariorum*: PRJNA553773, *A. pisum*: PRJNA13657, *A. craccivora:* PRJNA558689, *O. laevigatus:* PRJNA721944, *C. lectularius:* PRJNA167477, *R. prolixus:* PRJNA13648, *A. mellifera*: PRJNA471592, *N. vitripennis*: PRJNA575073, *F. occidentalis*: PRJNA203209, *T. palmi*: PRJNA607431, *Z. nevadensis:* PRJNA203242.

### Comparative genomics

The manually curated *S. rueppellii* detoxification genes were used to perform comparative analyses with close relatives, pollinators and crop pest species. Protein sequences for these genes are included in Additional file 2 and the similarity matrices used to identify likely recent tandem duplications are included in Additional file 3. These duplications are indicated in Figs. S2-S6 which show the phylogenetic trees of each of these detoxification families.

#### UDP-glycosyltransferases

UDP-glycosyltransferases (UGTs) are phase II detoxification enzymes which are involved in insecticide metabolism. The mechanisms of UGT-mediated resistance are for example based on the conjugation of P450-functionalized substrates. Their upregulation has been shown in resistant strains of *P. xylostella* [37] and they have been linked to diamide resistance in *Chilo suppressalis* [98] and neonicotinoid resistance in *Diaphorina citri* [99]. They also contribute to insecticide detoxification via the elimination of oxidative stress in *Apis cerana* [100].

We detected 46 UGTs in the *S. rueppellii* genome (Table 2), which are classified into 14 families as shown in Fig. S2 (UGT36, UGT37, UGT49, UGT50, UGT301, UGT302, UGT308, UGT314, UGT316, UGT430, UGT431, UGT432, UGT433, UGT435). Of these families, UGT430-435 are species specific to *S. rueppellii*, whilst all other families are present in at least one additional Diptera species [101].

**Table 2:** Numbers of annotated UDP glucosyltransferase genes found in *Sphaerophoria rueppellii* (this study), *Drosophila melanogaster* [102], *Anopheles sinensis, Aedes aegypti, Anopheles gambiae *[101], *Apis mellifera, Bombus impatiens, Bombus huntii *[103]*, Tetranychus urticae, Nilaparvata lugens, Acyrthosiphon pisum, Bemisia tabaci *[104], *Myzus persicae *[105], *Trialeurodes vaporariorum *[106] and *Thrips palmi *[107]

	***S. rueppellii*** + close relatives					Pollinators			Crop pests						
	Diptera					Hymenoptera			Acari	Hemiptera					
	***Sr***	***Dm***	***As***	***Aa***	***Ag***	***Am***	***Bi***	***Bh***	***Tu***	***Nl***	***Mp***	***Ap***	***Tv***	***Bt***	***Tp***
**Total**	46	35	30	32	23	2	8	2	81	20	101	72	55	76	17

The UGT genes are distributed across predicted chromosomes 1-5 (with the exception of 1 gene, which is located on a scaffold additional to the chromosome superscaffolds) and the majority (26) are on potential chromosome 2. 38 of the genes are located within clusters of 2-13 tandem UGT genes which generally consist of genes from the same UGT family. This indicates that a high degree of tandem duplication within the UGT gene family likely occurred in *S. rueppellii*.

39 out of 46 UGT genes belong to 7 of the UGT families (UGT308, UGT36, UGT49, UGT302, UGT430, UGT37 and UGT431), suggesting a significant lineage-specific expansion within these 7 families. There appears to be a greater degree of UGT expansion in *S. rueppellii* compared to other Dipteran species. For example, in the *Drosophila melanogaster* genome, expansion is only seen in 3 UGT families (UGT35, UGT303, UGT37). In the three mosquito species *Anopheles sinensis, Anopheles gambiae* and *Aedes aegypti* expansion is only seen in the UGT308 subfamily [101]. We further noted that *S. rueppellii* also has a much higher number of UGT genes compared to other pollinator species (Table 2).

Hemiptera crop pest species had higher numbers of UGT genes than Diptera (Table 2). This tends to be the result of substantial gene expansion concentrated within a single UGT family. For example: a UGT352 expansion in *Bemisia tabaci* accounted for 36 of its 76 UGTs; the UGT344 family accounted for 35 of *Acyrthosiphon pisum’s* 72 UGTs and the UGT201 family accounted for 33 of *Tetranychus urticae’s* 81 UGTs [108]. These expansions have previously been linked to increased detoxification of plant secondary metabolites, suggesting that the increased number of UGTs in Hemiptera compared to Diptera may be linked to differences in diet. Host plant adaptation alone has been shown to usually be insufficient to confer insecticide resistance, and therefore higher numbers of UGTs in Hemiptera cannot be assumed to correspond to increased insecticide tolerance/resistance [109]. However, upregulation of UGTs from 7 different UGT families, including 6 UGT344 members, has been associated with thiamethoxam resistance in *Aphis gossypii *[110]. It is therefore possible that expansion in UGT families may be associated with both host plant adaptation and insecticide resistance. Further study into the role of individual UGTs would be needed to clarify whether differences in total numbers of UGTs are associated with differences in insecticide tolerance/resistance between Hemiptera and Diptera.

Nine of the *S. rueppellii* UGT genes belonged to the UGT302 and UGT308 families, which are currently the families most associated with resistance to pyrethroid insecticides [101]. This suggests that expansion within these families in *S. rueppellii* could be a response to pyrethroid exposure. Expansions of these gene families have been reported in *A. sinensis*. Specifically, 14 of its 30 UGT genes belonged to the UGT302/308 families and 7 of these were considered strong candidates for pyrethroid resistance [101].

The most significant expansion was seen in the UGT431 family, which is unique to *S. rueppellii*. This family is closest in sequence similarity to the UGT37 and UGT430 families which were also expanded. The UGT37 family has been shown to be upregulated during organophosphorus pesticide exposure in *Caenorhabditis elegans* [111]. The UGT37 family exhibits lineage specific expansion in *D. melanogaster* and is its largest UGT gene family with members spread across five different genome locations [102]. This differs from the *S. rueppellii* genome, where the majority (12/14) of the UGT37 and UGT431 families are located in a cluster of adjacent genes on chromosome 2 within 0.17Mb of genomic space. This could suggest the UGT37 family may have expanded more recently in *S. rueppellii*. However, the percentage identity within this cluster ranges from 33% to 70%, which indicates that at least part of the cluster can be considered “old”. Since these genes have not been fully dispersed in the genome, there may be a selective advantage for preserving the cluster on chromosome 2 as a heritable unit, i.e. UGT37/431 members may be required for the same mechanism. Based on the links of UGT37 to pesticide resistance, the expansion of the UGT37/431 families and preservation of the gene cluster could be an adaptational response to pesticide exposure.

#### Glutathione *S*-transferases

The glutathione *S*-transferases (GSTs) family is large and functionally diverse, and has been shown to confer resistance to all main insecticide classes. For example, the delta and epsilon classes have been linked to pyrethroid resistance in *A. aegypti* and *N. lugens *[112, 113]. GST-mediated detoxification of insecticides takes place via several mechanisms, including protecting against oxidative stress, binding and sequestration of the insecticide and by catalysing the conjugation of glutathione to insecticides and their metabolites to reduce their toxicity and facilitate excretion, respectively [39].

*S. rueppellii* has 23 GSTs (Table 3), which are located on proposed chromosomes 1-3, with members of the same family located on the same chromosome. (Chr1: Theta and Omega, Chr2: Epsilon, Chr3: Sigma, Delta and Zeta.) The total number of GSTs is slightly lower in *S. rueppellii* compared to other Diptera species, although higher than other pollinators. A phylogenetic tree of these GSTs, including likely recent tandem duplications are included in Fig. S3.

**Table 3:** Numbers of GST genes annotated in *Sphaerophoria rueppellii *(this study), *Drosophila melanogaster* [114], *Aedes aegypti* [115], *Anopheles gambiae* [116], *Culex pipiens quinquefasciatus* [117]*, Apis mellifera, Bombus impatiens, Bombus huntii* [118], *Thrips palmi* [107], *Myzus persicae, Acyrthosiphon pisum, Trialeurodes vaporariorum* and *Bemisia tabaci* [119] and their distribution across classes

	***S. rueppellii +*** close relatives					Pollinator			Crop pests				
	Diptera					Hymenoptera			Hemiptera				
	***Sr***	***Dm***	***Aa***	***Ag***	***Cp***	***Am***	***Bi***	***Bh***	***Tp***	***Mp***	***Ap***	***Tv***	***Bt***
**Delta**	4	9	8	12	14	1	-	-	14	3	11	9	14
**Epsilon**	11	14	8	8	10	0	-	0	0	0	0	1	0
**Omega**	3	4	1	1	1	1	-	-	1	1	1	0	1
**Sigma**	1	1	1	1	1	4	-	3	6	12	5	3	6
**Theta**	3	4	4	2	6	1	-	-	1	1	2	0	0
**Zeta**	1	2	1	1	0	1	-	-	2	0	0	2	2
**Microsomal**	0	3	3	3	3	2	-	-	1	2	2	3	2
**Total**	**23**	**37**	**26**	**28**	**35**	**10**	**15**	**11**	**25**	**19**	**21**	**18**	**25**

Sigma-GSTs are associated with detoxification of oxidants produced during pollen and nectar metabolism in bees [120]. However, *S. rueppellii* has a reduced number of sigma-GSTs compared to other pollinators. This suggests *S. rueppellii* may use different detoxification enzymes to cope with these oxidants, or perhaps a different pathway for pollen and nectar metabolism.

Within the Diptera species the majority of GSTs are present within the epsilon and delta class, however, for *S. rueppellii* whilst the numbers of epsilon GSTs (11) are comparable to other Diptera species, the numbers of delta class GSTs (4) are notably lower.

The epsilon class is the largest class in *S. rueppellii*, as a result of substantial class-specific expansion. 7 epsilon members are clustered within 31kb, with a percentage identity ranging from 35% to 81%, this indicates that whilst some members of the cluster are the result of recent tandem duplications, others are the result of far older duplications. Clusters of epsilon GSTs are common across Diptera species, with clusters of 8 epsilon genes seen in *A. aegypti* and *A. gambiae* and a cluster of 11 epsilon genes in *D. melanogaster* [121]. The preservation of these clusters suggests that maintaining epsilon genes as a heritable cluster confers a selective advantage, likely in terms of conferring increased insecticide resistance. This cluster and class specific expansion may therefore imply an increased degree of GST delta-linked pyrethroid tolerance/resistance in *S. rueppellii* compared to Hemiptera crop pests, which have at most 1 epsilon gene.

In contrast to the epsilon class, *S. rueppellii’s* delta class is far smaller, as a result of minimal class-specific expansion. Only 2 of the genes are directly adjacent, and were likely a recent tandem duplication based on their 88% sequence identity, whilst the other two members are dispersed across 7.8Mb of genomic space. This follows the pattern seen in some other Diptera species, which also have delta genes more widely dispersed than epsilon. For example, 3 separate delta clusters are seen in both *A. aegypti* and *A. gambiae*, although in *D. melanogaster* a single cluster of 11 delta genes is present [121]. This reduced number of delta GSTs in *S. rueppellii* could imply a reduced degree of GST delta-linked pyrethroid resistance compared to Hemiptera crop pests, although this may be counteracted by the significant expansion within the epsilon class. The lack of preservation of delta clusters may also suggest that they confer a less significant selective advantage than do the epsilon GSTs.

The sigma class of GSTs has been associated with the detoxification of organophosphorus insecticides [122]. All Diptera species included in analysis had only 1 sigma gene, and this was also the case for *S. rueppellii*. All crop pest species had larger sigma classes. This may imply a reduced level of GST sigma-linked organophosphorus resistance compared to Hemiptera crop pests.

#### Carboxyl/choline esterases

Carboxyl/choline esterases (CCEs) are associated with insecticide resistance, notably to organophosphates, and to a lesser degree carbamates and pyrethroids [41]. For example esterase-based organophosphate resistance has been reported in three *Culex* species [123] and synergist bioassays have shown that esterases are responsible for metabolic resistance to pyrethroids (deltamethrin) and organophosphates (temephos) in *A. aegypti* [124].

*S. rueppellii* has 40 full-length carboxylesterase genes (Table 4) which are distributed across proposed chromosomes 1-5 with 19 of the genes arranged in 7 clusters of 2-4 genes (Fig. S4). The total number of CCEs for *S. rueppellii* and the distribution of genes across the 3 main classes is comparable to other Diptera species. The numbers and distribution of CCEs is also similar between Diptera and Hemiptera, with the only noticeable differences being a lower average number of ‘dietary’ esterases in Hemiptera species and a higher number of ‘glutactins’ in Diptera. Compared to other pollinators, *S. rueppellii* has a much higher number of CCE genes.

**Table 4:** Numbers of CCEs annotated in *Sphaerophoria rueppellii *(this study), *Drosophila melanogaster, Aedes aegypti, Anopheles gambiae* [125], *Culex pipiens quinquefasciatus* [117], *Apis mellifera, Bombus impatiens, Bombus huntii* [118], *Frankliniella occidentalis* [126], *Myzus persicae* [127], *Acyrthosiphon pisum, Bemisia tabaci* [128] and *Trialeurodes vaporariorum* [129] and their distribution across classes and clades

		***S. rueppellii*** and close relatives					Pollinators			Crop pests				
		Diptera					Hymenoptera			Hemiptera				
		***Sr***	***Dm***	***Cp***	***Aa***	***Ag***	***Am***	***Bi***	***Bh***	***Fo***	***Mp***	***Ap***	***Tv***	***Bt***
**Dietary class**		15	13	30	22	16	8	-	-	28	5	5	12	6
**Hormone/semiochemical processing class**		13	8	26	15	14	5	-	-	7	12	16	6	19
**Neuro- developmental class**	Glutactins	4	5	6	7	10	0	-	-	2	0	0	1	1
	AChE	1	1	1	2	2	2	-	-	2	3	2	2	4
	Uncharacterised	-	1	2	1	1	3	-	-	2	1	1	1	1
	Gliotactin	1	1	1	1	1	1	-	-	1	1	1	1	1
	Neuroligin	5	4	3	5	5	5	-	-	7	0	3	3	10
	Neurotactin	1	2	2	2	2	-	-	-	1	0	0	1	0
	**Subtotal**	12	14	15	18	21	11	-	-	15	5	7	9	17
**Total**		**40**	**35**	**71**	**55**	**51**	**24**	**22**	**23**	**50**	**22**	**28**	**27**	**42**

The so-called ‘dietary’ class of CCEs has been shown to be involved in insecticide and xenobiotic detoxification [125] and amplification of genes within this class, i.e. esterase E4/B1-like genes, has been linked to organophosphate resistance in hemipteran and dipteran species (*M persicae, N. lugens, S. graminum* and *Culex* mosquitoes) [123, 130–134]. Within the *S. rueppellii* genome, multiple clusters of high similarity, adjacent esterase E4/B1 genes indicate recent tandem duplications, which could confer some tolerance/resistance to organophosphorus insecticides. In cases where the number of dietary genes in *S. rueppellii* is higher than Hemiptera crop pests there could be an increased degree of organophosphate resistance.

#### ABC Transporters

ATP-binding cassette transporters (ABCs) are the largest known group of active transporters and are able to eliminate by translocation xenobiotic compounds such as secondary metabolites produced by plants or insecticides [38]. The ABC transporters are subdivided into eight subfamilies (ABCA-H), of which ABCB, ABCC and ABCG are the most associated with resistance to a variety of insecticides including pyrethroids, carbamates, organophosphates and neonicotinoids [135].

*S. rueppellii* has 47 ABC genes (Table 5), which are distributed across proposed chromosomes 1-6, with 3 of the genes located on scaffolds external to the chromosome superscaffolds. 20 of the genes are located in 9 clusters of 2-3 (Fig. S5). The total number of ABC genes in *S. rueppellii* is at the lower end of that seen for other Diptera species, for which the total numbers range from 47 to 70, as well as for Hemiptera crop pests, which range from 45 to 77. The total number was slightly higher than pollinator *A. mellifera* which had 41 ABC genes.

**Table 5:** Numbers of ABC transporter genes annotated in *Sphaerophoria rueppellii *(this study), *Drosophila melanogaster* [135]*, Bactrocera dorsalis* [136], *Anopheles gambiae, Culex pipiens quinquefasciatus* [137], *Apis mellifera* [138]*, Aedes aegypti* [139]*, Anopheles sinensis* [140], *Frankliniella occidentalis* [126], *Thrips palmi* [107], *Aphis gossypii* [141], *Trialeurodes vaporariorum* [142] *Diuraphis noxia* and *Bemisia tabaci* [143] and their distribution across subfamilies

	***S. rueppellii*** + close relatives							Pollinators	Crop pests					
	Diptera							Hymenoptera	Hemiptera					
	***Sr***	***Dm***	***Bd***	***Aga***	***Aa***	***As***	***Cp***	***Am***	***Fo***	***Tp***	***Dn***	***Ago***	***Tv***	***Bt***
**ABCA**	11 (12*)	10	7	8	10	10	9	3	3	3	3	4	3	8
**ABCB**	6 (7*)	8	7	5	5	5	5	5	5	4	6	5	9	3
**ABCC**	8	14	9	15	15	16	18	9	19	12	24	25	7	6
**ABCD**	3	2	2	2	2	2	2	2	2	2	3	2	4	2
**ABCE**	1	1	1	2	1	1	2	1	1	2	1	1	1	1
**ABCF**	3	3	3	3	3	3	3	3	3	3	3	4	3	3
**ABCG**	10	15	15	17	15	21	28	15	22	16	26	30	9	23
**ABCH**	3	3	3	3	4	3	3	3	13	7	11	0	9	9
**Total**	45 (47*)	**56**	**47**	**55**	**53 (62 with 9 in ABCJ)**	**61**	**70**	**41**	**70**	**49**	**77**	**71**	**45**	**55**

*including fragment genes >200bp

The distribution of *S. rueppellii’*s ABC genes across subfamilies is similar to other species, except for the ABCC and ABCG subfamilies, which are smaller in *S. rueppellii* than all other Diptera species and the majority of Hemiptera crop pests (Table 5). These are two of the families most associated with insecticide resistance [135], and so their reduced size suggests that ABC-mediated tolerance/resistance to insecticides could be lower in *S. rueppellii* compared to these other species.

The ABCA subfamily is expanded in Diptera, whilst the ABCH subfamily is expanded in Hemiptera. However these subfamilies do not have strong links to insecticide resistance, and so these differences would likely not contribute to any variation in tolerance/resistance levels.

The percentage identity of ABC genes within *S. rueppellii* ranges from 0%-71%, with the exception of one pair of genes with an identity of 89%. This suggests that there has been little recent lineage specific expansion within the *S. rueppellii* ABC transporter family. This is further supported by the numbers of the genes in the ABC subfamilies, which are either similar to or lower than other Diptera species. Any potential lineage-specific expansion seen in *S. rueppellii* is minimal, demonstrated by the small size of gene clusters. Species-specific and lineage-specific ABC expansions on a much larger scale have been reported in a variety of arthropods such as *Tribolium castaneum* and *Tetranychus urticae*, although whether these expansions contribute directly to increased insecticide resistance is not yet known [135].

#### Cytochrome P450 monooxygenases

Cytochrome P450 monooxygenases (P450s) are a diverse superfamily capable of metabolizing a huge variety of endogenous and exogenous substrates. In insects they are involved with growth and development, metabolism of pesticides and plant toxins as well as the production and metabolism of insect hormones and pheromones [144, 145]. P450s are associated with the resistance to insecticides from a variety of classes, including pyrethroids, carbamates and neonicotinoids and many examples of resistance are linked to upregulated P450s [146–149]. They are also linked to the activation of organophosphates and other pro-insecticides (a form of insecticide which is metabolized into an active form inside the host) [40] often as a result of downregulation [150, 151].

A total of 69 full-length P450 genes were identified in the *S. rueppellii* genome, as well as 4 P450 fragment genes (Table 6). These genes were named by Dr David Nelson using his in-house pipeline (Fig. S6) [86]. The total number of P450s varies widely between insect species, ranging from 44 for *Bombus huntii* to 196 for *C. pipiens*. *S. rueppellii* falls at the lower end of this range, however when compared to other dipteran species, this is mainly due to the reduced size of the CYP4 clan.

**Table 6:** Total numbers of Cytochrome P450 genes annotated in *Sphaerophoria rueppellii *(this study), *Musca domestica, Drosophila melanogaster* [152], *Anopheles gambiae, Aedes aegypti* [153], *Culex pipiens quinquefasciatus* [117], *Apis mellifera* [154], *Bombus impatiens, Bombus huntii* [103], *Frankliniella occidentalis, Thrips palmi* [126], *Myzus persicae, Acyrthosiphon pisum* [127], *Trialeurodes vaporariorum* [142] and *Bemisia tabaci* [155]

	***S. rueppellii*** + close relatives						Pollinator			Crop pests					
	Diptera						Hymenoptera			Hemiptera					
	***Sr***	***Md***	***Dm***	***Ag***	***Aa***	***Cp***	***Am***	***Bi***	***Bh***	***Fo***	***Tp***	***Mp***	***Ap***	***Tv***	***Bt***
**CYP2**	6	8	7	10	11	14	8	-	-	12	12	3	10	7	18
**CYP3**	34(37)*	65	35	41	80	88	31	-	-	22	26	63	33	41	76
CYP6	22	46	22	-	-	-	-	-	-	18	-	-	29	34	47
CYP9	2	7	5	-	-	-	-	-	-	0	-	-	0	0	0
Other	10	12	8	-	-	-	-	-	-	4	-	-	4	7	-
**CYP4**	15(16)*	55	33	45	58	83	4	5	2	37	42	48	32	25	73
**Mitochondrial**	14	18	11	9	9	11	6	-	-	10	11	1	8	7	4
**Total**	69(73)*	**146**	**86**	**105**	**158**	**196**	**49**	**49**	**44**	**81**	**91**	**115**	**83**	**80**	**171**

*Values in brackets represent total gene numbers including partial and fragment genes. For other species partial and fragment P450 genes were excluded in cases where they were listed as such - some may remain in the counts if official naming and curation had not taken place.

34 of the P450 genes have 55-97% identity to another sequenced P450, 38 have 40-55% identity, and 1 gene has <40% identity. 9 genes (CYP18A1, CYP301-304A1, CYP307A2, CYP314A1, CYP315A1 and CYP49A1) were classified as orthologs to P450s from *Lucilia cuprina, Ceratitis capitata* and *Musca domestica.* These genes are involved in a conserved pathway, found in all insects, for the essential growth hormone 20-hydroxyecdysone [156]. Orthologs were not present for other genes, likely because other P450s are involved in detoxification, and therefore vary during evolution based on the organism’s environment and adaptation.

The CYPome (the full complement of P450s in the genome) diversity value was 52%, based on the presence of 38 CYP subfamilies and 73 genes. The CYPome follows the pattern of other arthropods, with most CYP families having few genes, whilst only a few CYP families have many genes [154].

The majority of *S. rueppellii* P450s (34) belong to the CYP3 clan, which is the one most associated with insecticide resistance, notably the CYP6 and CYP9 families [145], both of which were present in *S. rueppellii*. CYP3 is also the largest clan in other pollinators and in several other Diptera species and hemipteran crop pest species (Table 6).

**Fig. 5:**
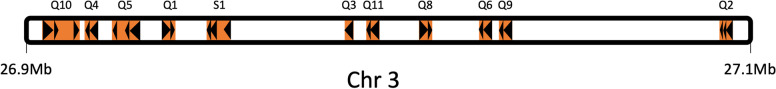
Arrangement of the CYP6Zx subfamily on chromosome 3. Orange boxes represent genes, black arrows represent exons as well as gene orientation

The largest sub-family specific expansion is in clan 3, within the CYP6Zx family, with 16 members: CYP6ZQ1-11, CYP6ZR1-4 and CYPZS1 (Fig. S6). Of these, CYP6ZQ1-11 (excluding Q7) are located contiguously within a 0.2Mb region of potential chromosome 3 (Fig. 5). Within this cluster there is no consistent relationship or pattern between the proximity of the CYP6Zx genes or their gene structure with their percent identity, which ranged from 33-90%. The lower end of the percent identity within the cluster indicates that at least part of the cluster can be considered “old”, and therefore, since these genes have not been fully dispersed in the genome, there may be a selective advantage for preserving the cluster on chromosome 3 as a heritable unit.

Whether the large CYP6Zx expansion may confer an increased degree of tolerance to xenobiotics in *S. rueppellii* remains to be investigated. Overall, numbers of the resistance-associated CYP3 clan are similar or lower than Hemiptera crop pests, suggesting that P450-mediated insecticide tolerance/resistance mechanisms may not be as prevalent as for other species.

The CYP4 clan is vastly expanded in many arthropods [157]. Whilst the CYP4 clan is not as strongly associated with insecticide resistance as CYP3, studies have shown upregulation of some CYP4 genes in response to insecticide exposure [147, 158–160]. *S. rueppellii* has a lower number of CYP4 genes compared to many other dipteran species and crop pests. However, compared to other pollinators the CYP4 subfamily is relatively large. A reduced number of CYP4 genes is common within pollinators [103, 161], but the reasons behind this are not yet known.

Pollinators use P450s for the detoxification of pollen flavonoids, notably the CYP6AS subfamily which is often expanded in honey bees [162, 163]. However, this subfamily is absent in *S. rueppellii*. It is likely that another subfamily is responsible for flavonoid detoxification in *S. rueppellii* (possibly the expanded CYP6Zx subfamily) and future studies assessing P450 upregulation in response to flavonoids could help identify this.

## Conclusions

Here we present the first high quality genome draft of *S. rueppellii* as well as its mitochondrial genome enabled by PacBio long-read technology combined with low error-rate short-read Illumina sequencing. Hi-C data permitted further scaffolding of this genome to a near-chromosome level assembly. A high completeness of 96% confirms the genome is of excellent quality for comparative and functional genomics analyses and provides a useful first reference for predatory syrphidae.

Comparative analyses of *S. rueppellii* with crop pests showed evidence that *S. rueppellii* has a detoxification gene inventory comparable to selected crop pests, with a few notable differences: potential lineage-specific expansions were seen within detoxification gene families such as UGTs and P450, whereas the ABC transporter family lacks such expansions compared to some crop pests. These expansions would need further analysis using close relatives to ensure they are not a product of the birth and death evolution with constant rates.

Comparative analyses of *S. rueppellii* with pollinators showed that *S. rueppellii* has an increased number of genes in all detoxification families, in particular: UGTs, non-sigma class GSTs and CYP4 P450s. This could be in part due to *S. rueppellii* needing more detoxification genes for its diet: hoverflies lack the eusocial behavioural mechanisms seen in bees, such as processing nectar into honey and converting pollen into ‘beebread’, which result in a dilution of toxins and hence reduce the need for detoxification enzymes in bees [161]. Additionally, the considerably longer migratory distance covered by hoverflies compared to bees [21] may have resulted in hoverflies being exposed to a wider variety of xenobiotics, and could perhaps have resulted in expansion of associated detoxification genes.

Despite the reduced number of detoxification genes in pollinators such as *A. mellifera*, they appear to be no more sensitive to insecticides than other insects [161, 164]. Insects with a pollen-based diet have been found to exhibit an increased degree of insecticide tolerance, with many of the same genes being upregulated in response to both pollen and to certain insecticides [165]. This suggests that the unique set of detoxification genes required by pollinators for their diet, could perhaps impart an increased degree of insecticide tolerance without the need for the extent of gene expansion seen in other insect species. This may mean that despite *S. rueppellii* having fewer detoxification genes than some crop pests, this might not necessarily be indicative of reduced insecticide tolerance. However, this is not to say that insecticides are not a major problem for *S. rueppellii*, with clear evidence that the same neonicotinoids (imidacloprid and thiamethoxam) which are toxic to honey bees are also toxic to *S. rueppellii* [166, 167].

This study provides a good basis for beginning to identify differences in genes encoding potential tolerance/resistance mechanisms between crop pests and *S. rueppellii* which could be exploited when selecting targeted insecticides for use in IPM strategies. Evidence of gene expansions in resistance-associated gene families implies that *S. rueppellii* is certainly capable of developing resistance to a variety of insecticides, which could be used to our advantage through the selective breeding and selection of resistant strains of *S. rueppellii* for use in IPM.

An interesting future comparison could be to look at the differences in olfactory genes between *S. rueppellii* and *E. pertinax* (the non-predatory European hoverfly), as this may give some indication of the mechanisms which enable *S. rueppellii* adults to locate aphid colonies for oviposition whilst avoiding parasitised aphids [14, 15].

## Supplementary Information

**Additional file 1.** Tables S1-S3 and figures S1-S6.

**Additional file 2. **Protein sequences of *Sphaerophoria rueppellii *insecticide resistance genes. 1. ABCs, 2. P450s, 3. CCEs, 4. GSTs, 5. UGTs.

**Additional file 3. **Similarity matrices for detoxification gene families in Sphaerophoria rueppellii. 1. ABCs, 2. P450s, 3. CCEs, 4. GSTs, 5. UGTs.

## Data Availability

The genome and transcriptome assemblies generated in this study (as well as the raw sequencing data used to produce them) are available under bioproject: PRJEB48036. The manually curated *Sphaerophoria rueppellii* genes used for comparative analyses are included in Additional file 2. The alignments and trees generated in this study are available in TreeBASE through the following link: http://purl.org/phylo/treebase/phylows/study/TB2:S29209.
