# Supplementary material for: A near-chromosome level genome assembly of the European hoverfly, Sphaerophoria rueppellii (Diptera: Syrphidae), provides comparative insights into insecticide resistance-related gene family evolution
Source: BMC Genomics. 2022 Mar 12;23:198. doi: 10.1186/s12864-022-08436-5 (PMC8917705; doi:10.1186/s12864-022-08436-5)
Supplement: Supplementary file 1 — Additional file 1. Tables S1-S3 and figures S1-S6. [file 12864_2022_8436_MOESM1_ESM.docx]

**Table S1. Statistics of Illumina short-read data mapped to the final *Sphaerophoria rueppellii* genome.** Values were obtained using samtools flagstat.

|  | **Total** | **Unique** |
| --- | --- | --- |
| **Total** | 834,971,684 | 625,569,153 |
| **Mapped** | 824,574,227 (98.75%) | 625,569,153 (100.00%) |
| **Properly paired** | 755,737,898 (93.16%) | 625,569,153 (100.00%) |
| **With itself and mate mapped** | 798,992,316 | 625,569,153 |
| **With mate mapped to a different scaffold** | 32,887,516 | 0 |

**Figure S1. Coverage of Illumina short-read data mapped to the genome.** Bigwig track produced using bamCoverage from deepTools and visualised in IGV.

**
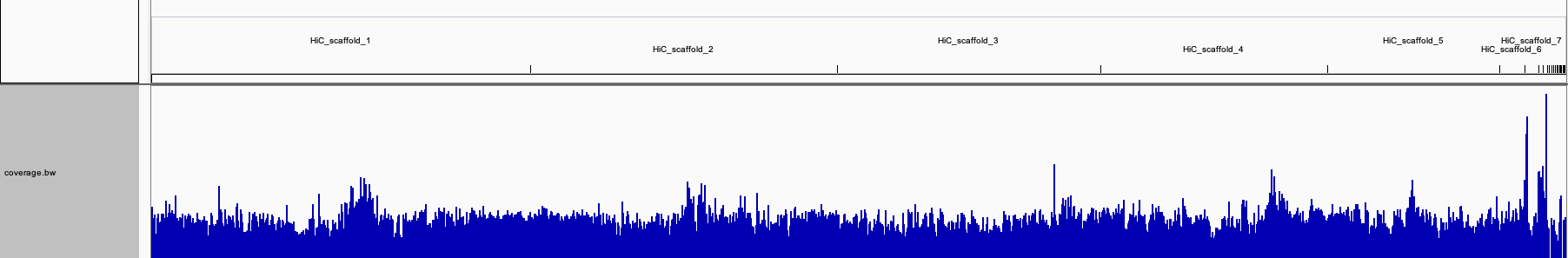
**

**
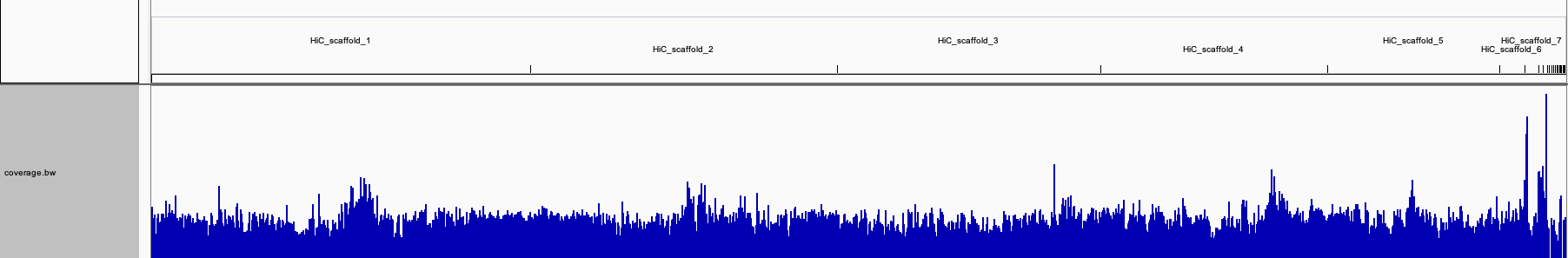
**

**Table S2. Number of ncRNAs predicted in the *S. rueppellii* genome**

| **ncRNA element** | **Number of elements** |
| --- | --- |
| tRNA | 2,058 |
| rRNA | 37 |
| snRNA | 79 |
| miRNA | 81 |
| srpRNA | 7 |
| snoRNA | 28 |
| lncRNA | 2 |

**Table S3. Summary of transposable and repetitive elements in the *S. rueppellii* genome**

Percentages do not include runs of X/Ns >=20

|  | | | **Number of Elements** | **Length Occupied (bp)** | **Percentage of Sequence** |
| --- | --- | --- | --- | --- | --- |
| **Retroelements** | | | 83,508 | 57,419,755 | 11.94 |
|  | **SINES** | | 21 | 6045 | 0.00 |
|  | **Penelope** | | 114 | 59,699 | 0.01 |
|  | **LINES** | | 75,547 | 47,912,743 | 9.97 |
|  |  | L2/CR1/Rex | 10,835 | 4,939,603 | 1.03 |
|  |  | R1/LOA/Jockey | 659 | 459,946 | 0.10 |
|  |  | R2/R4/NeSL | 37 | 26,828 | 0.01 |
|  |  | RTE/Bov-B | 56,568 | 39,492,974 | 8.22 |
|  | **LTR elements** | | 7,490 | 9,500,967 | 1.98 |
|  |  | BEL/Pao | 1,433 | 2,121,736 | 0.44 |
|  |  | Ty1/Copia | 2,122 | 2,534,993 | 0.53 |
|  |  | Gypsy/DIRS1 | 3,935 | 4,844,238 | 1.01 |
| **DNA transposons** | | | 16,586 | 8,092,263 | 1.68 |
|  | hobo-Activator | | 88 | 35,361 | 0.01 |
|  | Tc1-IS630-Pogo | | 16,004 | 7,819,876 | 1.63 |
|  | PiggyBac | | 27 | 8,274 | 0.00 |
|  | Other (Mirage, P-element, Transib) | | 23 | 13,960 | 0.00 |
| **Rolling-circles** | | | 3,616 | 462,376 | 0.10 |
| **Unclassified** | | | 6,299 | 3,448,721 | 0.72 |
| **Total interspersed repeats** | | | - | 68,960,739 | 14.35 |
| **Small RNA** | | | 54 | 15,691 | 0.00 |
| **Satellites** | | | 11 | 817 | 0.00 |
| **Simple repeats** | | | 150,503 | 6,316,344 | 1.31 |
| **Low complexity** | | | 36,195 | 1,890,143 | 0.39 |
| **Total** | | | **296,772** | **146,606,849** | **30.00** |

**
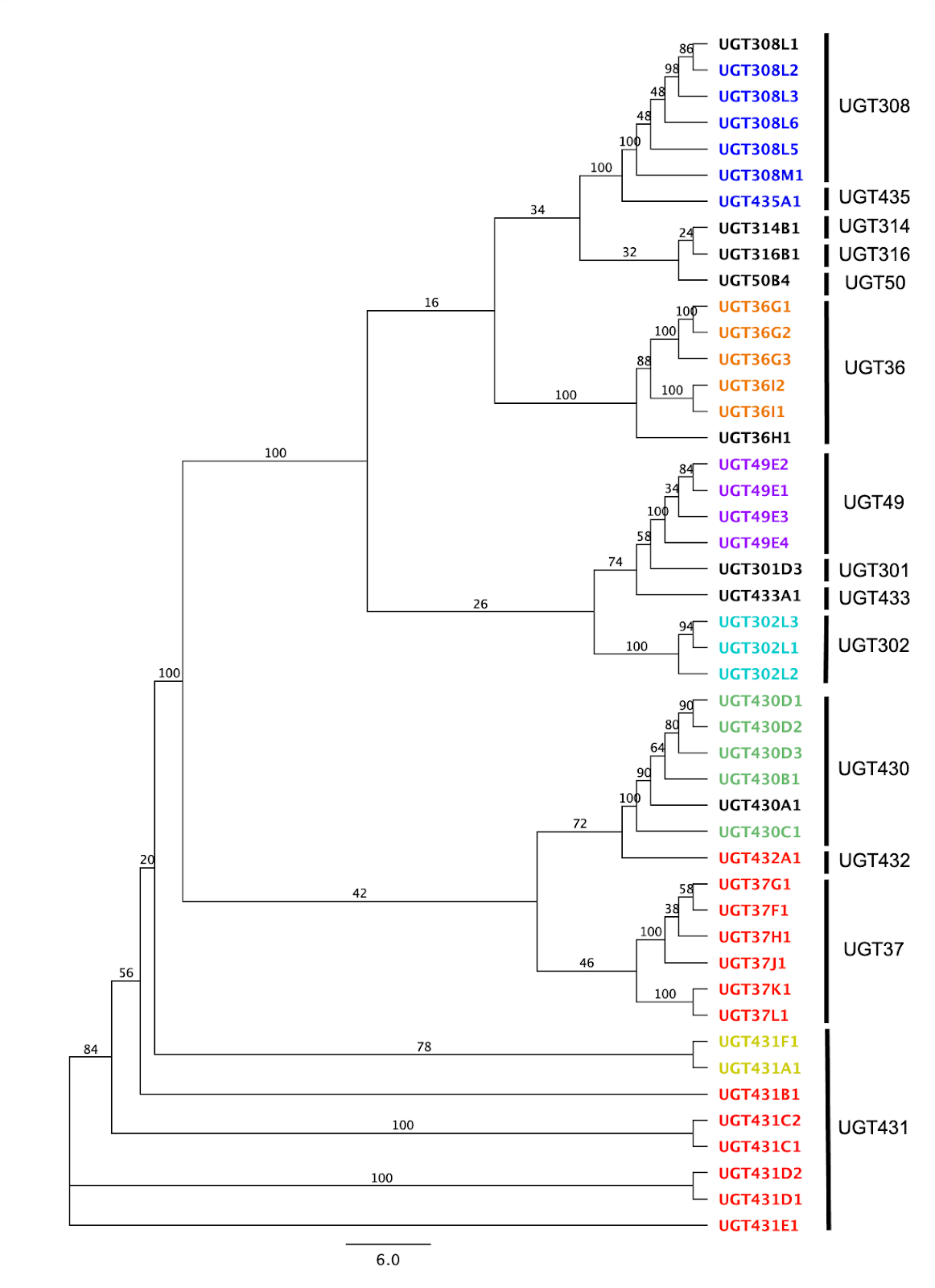
**

**Figure S2. Phylogenetic tree of *S. rueppellii* UDP-glycosyltransferases. Amino acid sequences were aligned using MAFFT and analyzed using RAxML (the GAMMA LG protein model was used). The bootstrap consensus tree was inferred from 100 replicates.** Coloured nodes indicate groups of likely recent tandem duplications, based on genes within the cluster having >70% similarity using Blosum45 with threshold 0, and being located adjacently in the genome.

**
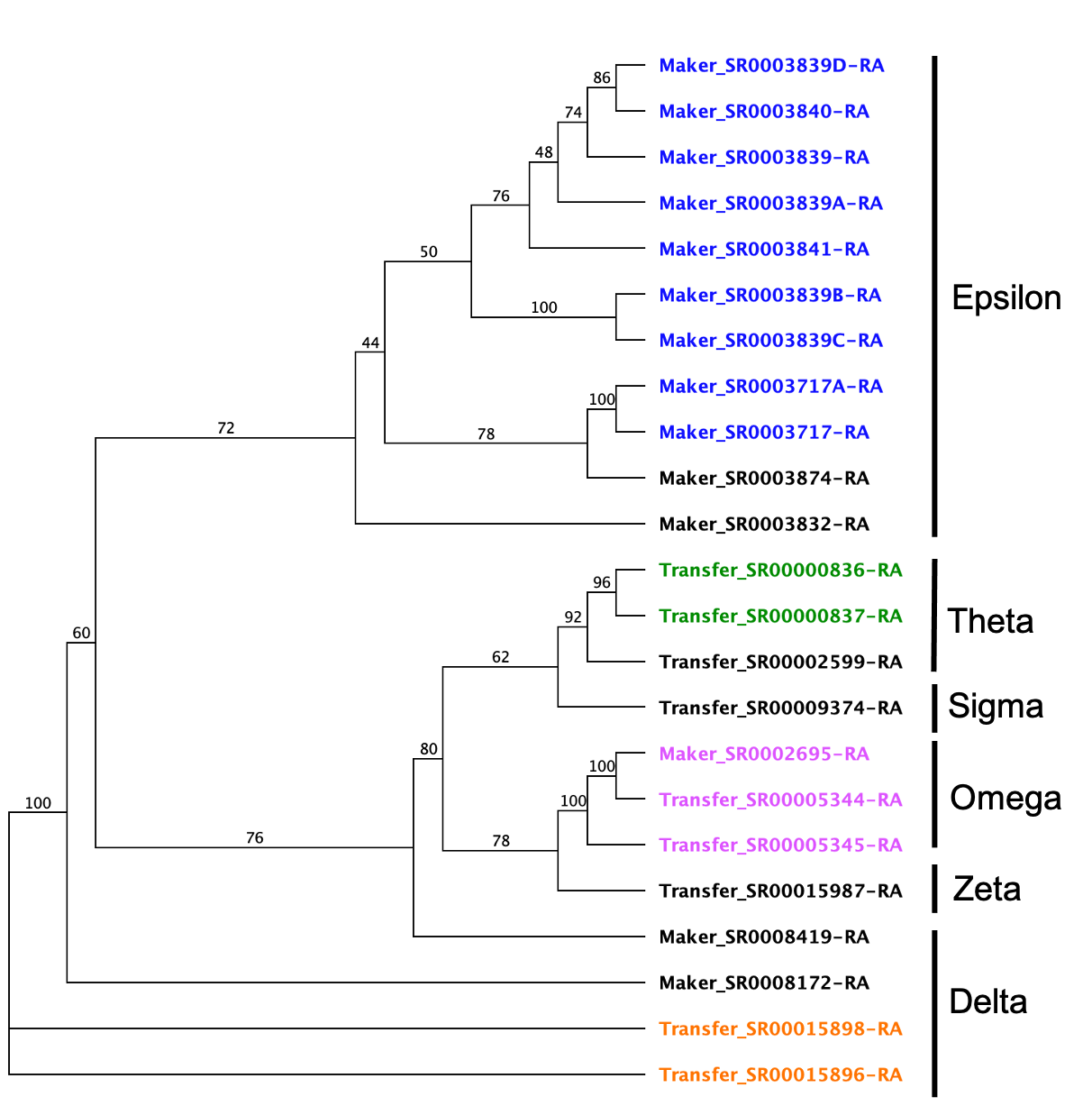
**

**Figure S3. Phylogenetic tree of the *Sphaerophoria rueppellii* glutathione S-transferases. Amino acid sequences were aligned using MAFFT and analyzed using RAxML (the GAMMA LG protein model was used). The bootstrap consensus tree was inferred from 100 replicates.**

Coloured nodes indicate groups of likely recent tandem duplications, based on genes within the cluster having >70% similarity using Blosum45 with threshold 0, and being located adjacently in the genome.

**
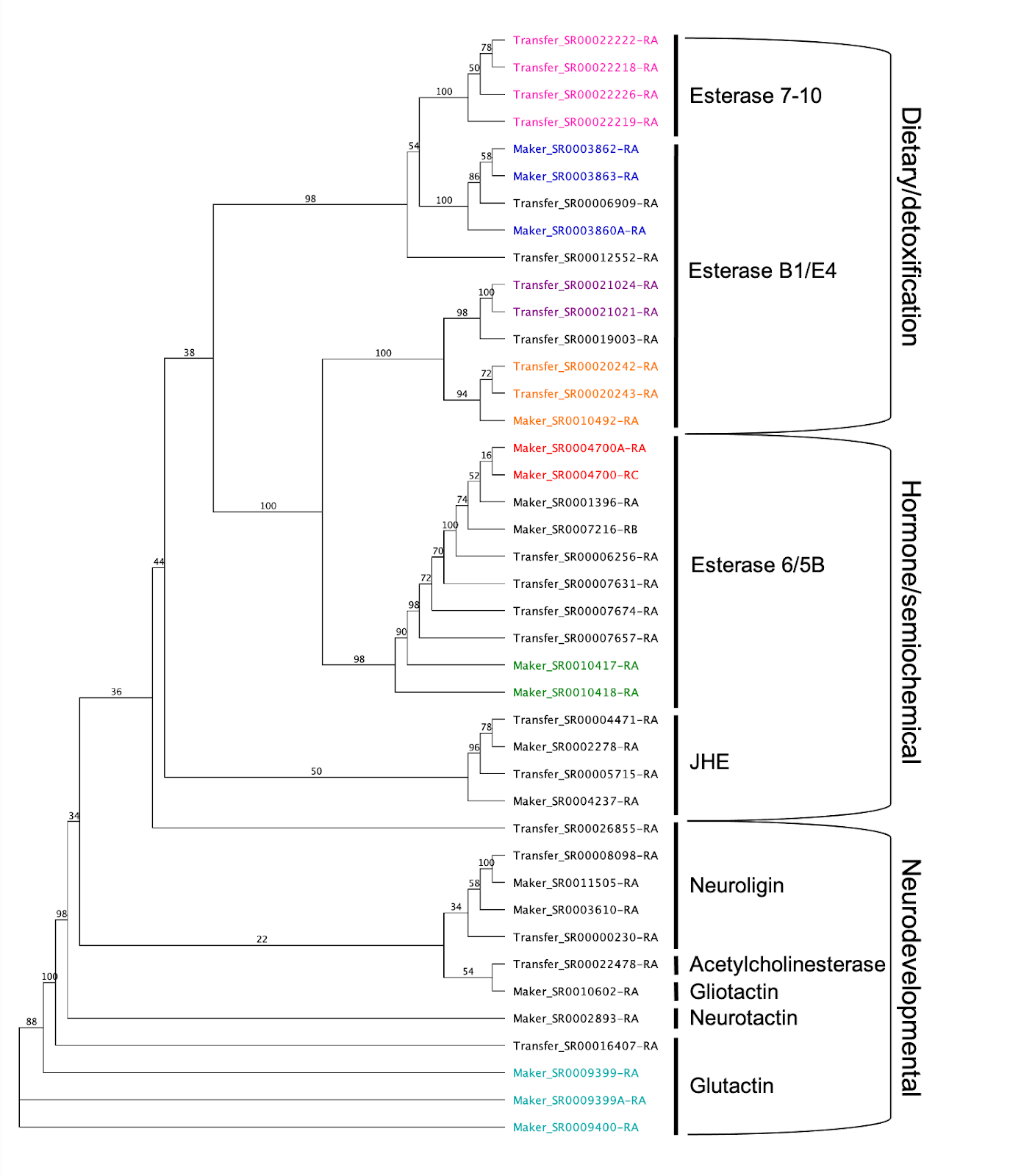
**

**Figure S4. Phylogenetic tree of the *Sphaerophoria rueppellii* carboxyl/cholinesterases. Amino acid sequences were aligned using MAFFT and analyzed using RAxML (the GAMMA LG protein model was used). The bootstrap consensus tree was inferred from 100 replicates.**

Coloured nodes indicate groups of likely recent tandem duplications, based on genes within the cluster having >70% similarity using Blosum45 with threshold 0, and being located adjacently in the genome. Maker_SR0001396-RA was a gene fragment, and was not included in the final gene count or analysis; all others are full-length genes.


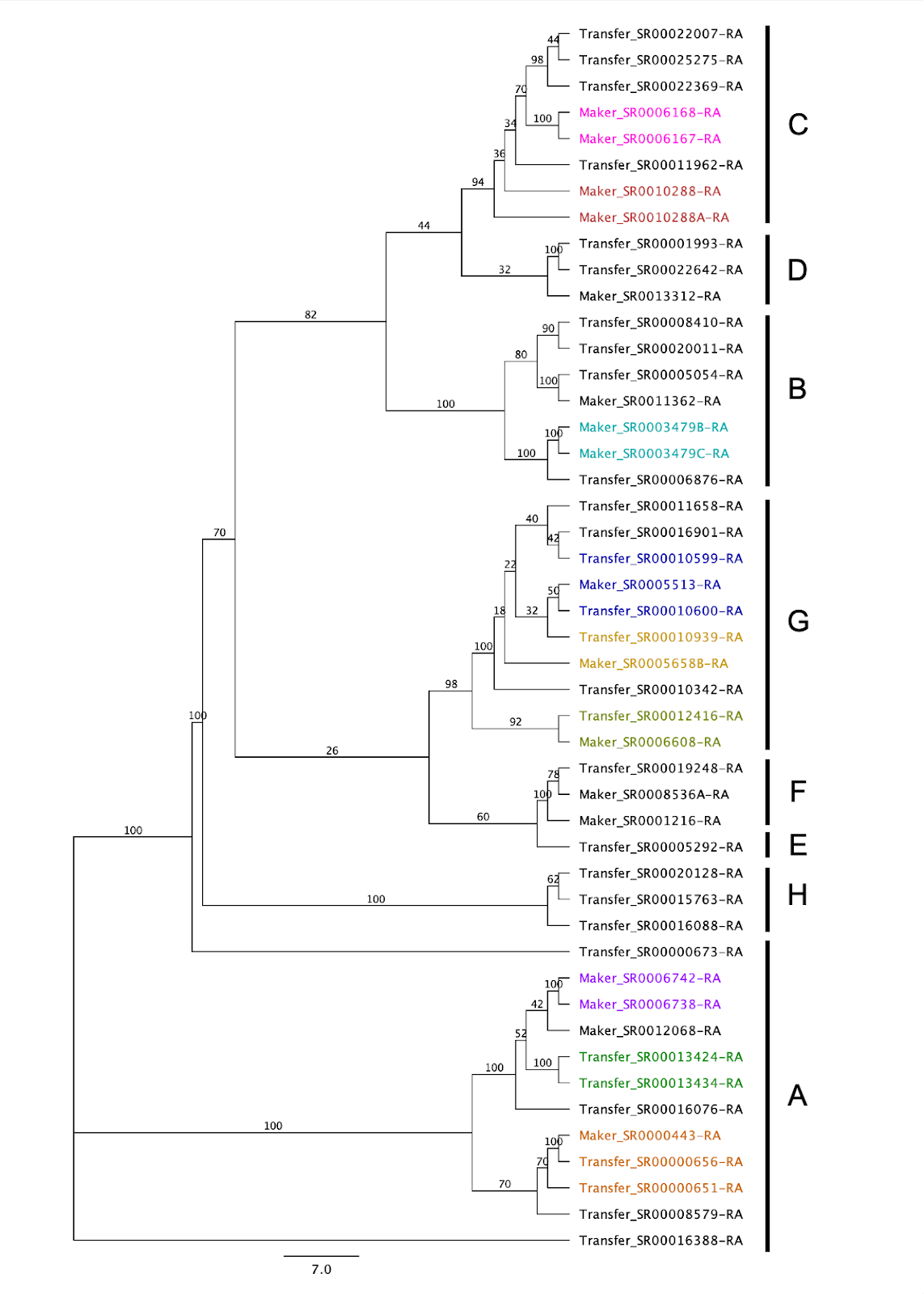


**Figure S5. Phylogenetic tree of the *Sphaerophoria rueppellii* ABC transporters. Amino acid sequences were aligned using MAFFT and analyzed using RAxML (the GAMMA LG protein model was used). The bootstrap consensus tree was inferred from 100 replicates.** Coloured nodes indicate groups of likely recent tandem duplications, based on genes within the cluster having >70% similarity using Blosum45 with threshold 0, and being located adjacently in the genome.

**Figure S6. Phylogenetic tree of the *Sphaerophoria rueppellii* Cytochrome P450s. Amino acid sequences were aligned using MAFFT and analyzed using RAxML (the GAMMA LG protein model was used). The bootstrap consensus tree was inferred from 100 replicates.** Coloured nodes indicate groups of likely recent tandem duplications, based on genes within the cluster having >70% similarity using Blosum45 with threshold 0, and being located adjacently in the genome. The CYP6Zx family is part of clan 3
